# Risk, reward and loss in addictive behavior: a six-year cross-lagged panel study

**DOI:** 10.1038/s41598-025-17826-0

**Published:** 2025-08-30

**Authors:** Anja Kräplin, Mohsen Joshanloo, Juliane Hilde Fröhner, Christian Baeuchl, Gerhard Bühringer, Thomas Goschke, Michael N. Smolka

**Affiliations:** 1 Department of Psychiatry and Psychotherapy, Technische Universität Dresden, Dresden, Germany; 2 Faculty of Psychology, Technische Universität Dresden, Dresden, Germany; 3 Department of Psychology, Keimyung University, Daegu, South Korea; 4 IFT Mental Health Solutions, Munich, Germany

**Keywords:** Decision-making, Risk, Impulsivity, Addictive behaviors, Longitudinal, Cross-lagged panel, Psychology, Human behaviour

## Abstract

Decision making in the context of addiction is characterized by altered values regarding risk and reward, but the long-term and reciprocal relationship between value-based decision-making and addictive behavior remains poorly understood. In this study, 338 individuals (19–27 years, 59% female) from a community sample participated in a baseline assessment, including clinical interviews on addictive behaviors (quantity of use, frequency of use, DSM-5 criteria) and a task battery to assess four facets of value-based decision-making (delay aversion, risk-seeking for gains and losses, and loss aversion). Follow-up assessments were conducted after 3 and 6 years, with 75% and 71% of participants retained, respectively. Using random-intercept cross-lagged panel models (RI-CLPM) controlled for age, gender, IQ, and the baseline addiction level, we examined the bidirectional relationships between value-based decision-making and addictive behavior. Our findings demonstrate temporal stability in both decision-making and addictive behavior, but provide limited evidence for predictive cross-lagged relationships. While prior research indicates that altered value-based decision-making is a predictor of addictive behavior, our findings suggest that its impact may be more limited or context-dependent, highlighting the importance of multifaceted approaches to understanding addiction.

**Supplementary Information:**

The online version contains supplementary material available at 10.1038/s41598-025-17826-0.

## Introduction

Addictive behavior is frequently characterized by impulsive decision-making, where individuals prioritize immediate rewards, such as pleasure or stress relief, over long-term health, social, and financial benefits. Impulsivity in value-based decision-making, including a heightened focus on short-term outcomes, has been identified as a core feature of addiction^1,2^. This paper aims to investigate the longitudinal and causal relationships between four distinct facets of value-based decision-making and addictive behavior, defined broadly to include both the quantity and frequency of substance use^3^ and the severity of addictive disorders, with or without substance involvement^4^.

Value-based decision-making is a multidimensional construct that has been conceptualized in different ways across studies^5^. To better capture addiction-related changes, we focus on four critical facets and corresponding alterations in addiction: (i) higher delay aversion, (ii) higher risk-seeking for uncertain gains, (iii) lower risk-seeking for uncertain losses, and (iv) lower aversion to potential losses^6–8^. These facets reflect core cognitive and emotional processes that are altered in addiction, including impaired cognitive control and reward and punishment processing^9^. Previous research from our lab has used a comprehensive value-based decision-making battery to assess these domains^10^ demonstrating that decision-making deficits are significantly cross-sectionally and longitudinally associated with addictive behavior^11–13^.

While decision-making alterations are increasingly recognized as central to addictive behavior^1,14^ the longitudinal predictive relationship between specific facets of decision-making and addictive behavior remains poorly understood. Two reasons are particularly noteworthy in this regard. First, existing longitudinal studies have focused on delay discounting, where steeper discounting (the preference for smaller, immediate monetary rewards over larger, delayed monetary rewards) has been shown to predict smoking initiation^15^ and alcohol use in adolescence^16,17^. However, other studies have found no evidence for a predictive association between delay aversion and future alcohol use^18^. The role of decision-making facets beyond delay aversion, such as risk-taking for uncertain gains or losses and loss aversion, remains insufficiently explored in the context of addiction. This is crucial, as decisions related to addictive behavior involve not only immediate and delayed rewards but also risks and negative consequences (e.g., health problems). In two studies from our lab (one with the same baseline sample as in this paper), we found that higher delay aversion, higher risk-seeking for gains, lower risk-seeking for losses and lower loss aversion predicted increased addictive disorder severity^11,12^.

A second reason for the lack of progress in research on the longitudinal role of value-based decision-making in addictive behavior is the use of inadequate statistical models, e.g., traditional cross-lagged panel models are limited by their inability to separate within-person fluctuations from stable between-person differences, often conflating individual variability with time-invariant traits^19^. To address this research need, our study employs random intercept cross-lagged panel models (RI-CLPM), a powerful analytical approach that offers distinct advantages for examining longitudinal relationships. The RI-CLPM accounts for stable between-person differences through random intercepts, thus isolating the within-person changes that are critical for understanding how alterations in decision-making influence addictive behavior over time. This approach allows for a more nuanced investigation of causal relationships, providing clearer insights into whether changes in decision-making drive addiction or vice versa.

Building on our theoretical model and prior work, we hypothesize that alterations in value-based decision-making, including higher delay aversion, higher risk-seeking for gains, lower risk-seeking for losses and lower loss aversion, increase the likelihood of addictive behavior. Consistent with the arguments of other researchers for delay aversion, we propose that altered value-based decision-making accounts for common symptoms across diagnostic categories and acts as a transdiagnostic process^20,21^.

In addition, we propose that addictive behavior, particularly severe or chronic use, may further exacerbate decision-making alterations. Emerging evidence indicates that addiction-related neuroadaptations, including changes in the brain’s reward system, impair cognitive control and decision-making processes, particularly in younger individuals^22,23^. For instance, one study from our lab demonstrated that higher internet gaming disorder severity predicted greater delay aversion one year later^24^.

Using RI-CLPMs, we aim to disentangle the bidirectional and potentially reciprocal relationships between decision-making and addictive behavior. This approach allows us to determine whether specific decision-making alterations predict the onset or escalation of addictive behavior, or whether addictive behavior contributes to changes in decision-making processes. Ultimately, this research will advance our understanding of the cognitive underpinnings of addiction and highlight potential intervention targets that could mitigate both decision-making impairments and addictive behaviors.

## Methods

### Design and procedure

We report how we determined our sample size, all data exclusions, all manipulations, and all measures in this study. The data of the study was collected within the project “Volitional Dysfunction in Self-control Failures and Addictive Behaviors,” part of the Collaborative Research Centre SFB 940 “Volition and Cognitive Control” at Technische Universität Dresden, Germany (Study protocol: ClinicalTrial.gov NCT04498988, OSF link: https://osf.io/yu5rm/). The study has an observational, prospective-longitudinal cohort design with seven measurement points. Baseline assessment involved four components: (1) clinical assessment, (2) ecological momentary assessments (EMA) of self-control failures in daily life, (3) cognitive control and decision-making task battery, and (4) MRI-based neuroimaging. Follow-ups occurred annually after the baseline MRI, with comprehensive evaluations at 3- and 6-year intervals and abbreviated clinical assessments during other years. In this paper, we analyzed the bidirectional relationship between value-based decision-making and addictive behavior over time, with data collected at baseline, 3-year follow-up, and 6-year follow-up. Previous publications using data from our sample have mainly focused on two areas: the prediction of self-control failures based on neurocognitive traits^25–29^ and the prediction of addictive behaviors based on neurocognitive traits and self-control failures^11,30,31^. One of our studies examined group differences in value-based decision-making between individuals with addictive behaviors and a control group, as well as the prediction of addictive behavior one year later^11^. The present study extends this research by incorporating a longer observation period of six years and employing cross-lagged panel models to disentangle the directional relationships and identify predictors over time. The study was conducted in accordance with the Declaration of Helsinki and approved by the Ethics Committee of the Technische Universität Dresden. Informed consent was obtained from all participants prior to their participation in the study.

### Recruitment and participants

**Fig. 1:**
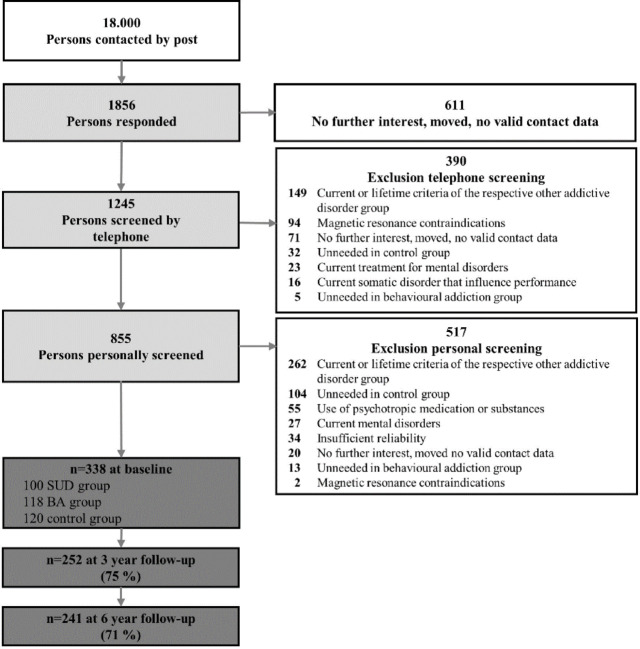
Flow chart of participants at baseline recruitment and over the six years of the study. Note. Reproduced from Kräplin et al. (2024), licensed under CC BY^30^. SUD = substance use disorder; BA = behavioral addiction.

Between 2013 and 2016, 18,000 randomly selected individuals aged 19–27 from Dresden, Germany, were invited to participate via postal mail. A response rate of 10.3% yielded 1,854 initial contacts. In total, 855 participants were invited for a face-to-face diagnostic screening. Based on the overarching study objective of investigating transdiagnostic processes, as well as the aim to obtain an inhomogeneous sample and include only legal behaviors due to ethical considerations, we focused on six dependent behaviors: alcohol use, tobacco use, gambling, internet use, gaming, and shopping. Exclusion criteria included difficulty providing informed consent, disorders affecting cognition, MRI contraindications, current mental health treatment, and psychotropic medication or substance use. After a personal screening using the Munich-Composite International Diagnostic Interview (DIA-X/M-CIDI)^32^, 338 participants met all inclusion criteria (Fig. 1). The sample size calculation is presented in Sect. 1 of the supplemental material. The baseline sample was divided into three groups:

1. **Substance use disorder group**: Participants met two or more DSM-5 criteria for alcohol and/or tobacco use disorders in the past 12 months, without lifetime behavioral addiction (*n* = 100).

2. **Behavioral addiction group**: Participants met two or more DSM-5 criteria for gambling or addictive disorders related to internet use, gaming, or shopping, with no history of Substance Use Disorders (*n* = 118).

3. **Control group**: Participants had no lifetime diagnoses of Substance Use Disorders or Behavioral Addictions (*n* = 120).

The participation rates of the clinical interviews in which we assessed addictive behavior declined over time due to participant dropout, with 75% (*n* = 253) of the baseline sample completing the clinical assessment at the 3-year follow-up and 71% (*n* = 235) at the 6-year follow-up. At the baseline, 335 participants took part in the task battery to assess value-based decision-making (3 were missing due to technical problems), 73% (*n* = 247) of the population took part in the 3-year follow-up and 54% (*n* = 182) in the 6-year follow-up.

### Measurements

#### Addictive behaviors

At baseline and each follow-up, addictive behaviors were assessed using a modified version of the Munich-Composite International Diagnostic Interview (DIA-X/M-CIDI)^32^. This structured interview evaluated the DSM-5 criteria for substance use disorders (alcohol and tobacco) and behavioral addictions (gambling, internet use, gaming, and shopping). The assessment included the quantity of use (e.g., grams of ethanol, cigarettes smoked, or hours spent on behavioral activities) and frequency of use (e.g., days per week). To capture the severity and impact of addictive behaviors, three indices were created by standardizing and aggregating the data across time points:

1. **Quantity of use**: Amount consumed (e.g., grams of alcohol, number of cigarettes, or hours spent online). The different values for quantity of use were normalized (i.e., rescaled to a range of 0 to 1) to ensure positive values for later additions. Normalization was carried out in a long data format to make the values comparable across different addictive behaviors and time points (baseline and follow-ups). Then, the values were summed up to form a composite score at each time point (range: 0–6 for six addictive behaviors).

2. **Frequency of use**: Measured in days per week (0 to 7 days). The frequencies of use across the six addictive behaviors were summed up into a composite score at each time point (range: 0–42).

3. **Addictive disorder severity**: DSM-5 criteria were used for substance-related and gambling disorders. We adapted the 11 DSM-5 Substance Use Disorder criteria to assess diagnostic criteria for behavioral addictions not currently included in the DSM-5 (i.e., internet, gaming, and shopping disorders). For example, the criterion ‘persistent desire or unsuccessful efforts to cut down or control substance use’ was rephrased as ‘Have you ever tried unsuccessfully to limit your computer gaming for a few days?’ This procedure was applied consistently across all non-substance-related behaviors. As with Substance Use Disorder, participants were considered to meet the threshold for a behavioral addiction if they endorsed two or more of the 11 adapted criteria. The number of criteria met per behavior was summed and used as an indicator of the addictive disorder severity. Summed scores ranged from 0 to 64, depending on the number of behaviors and criteria fulfilled.

#### Value-based decision-making

**Fig. 2:**
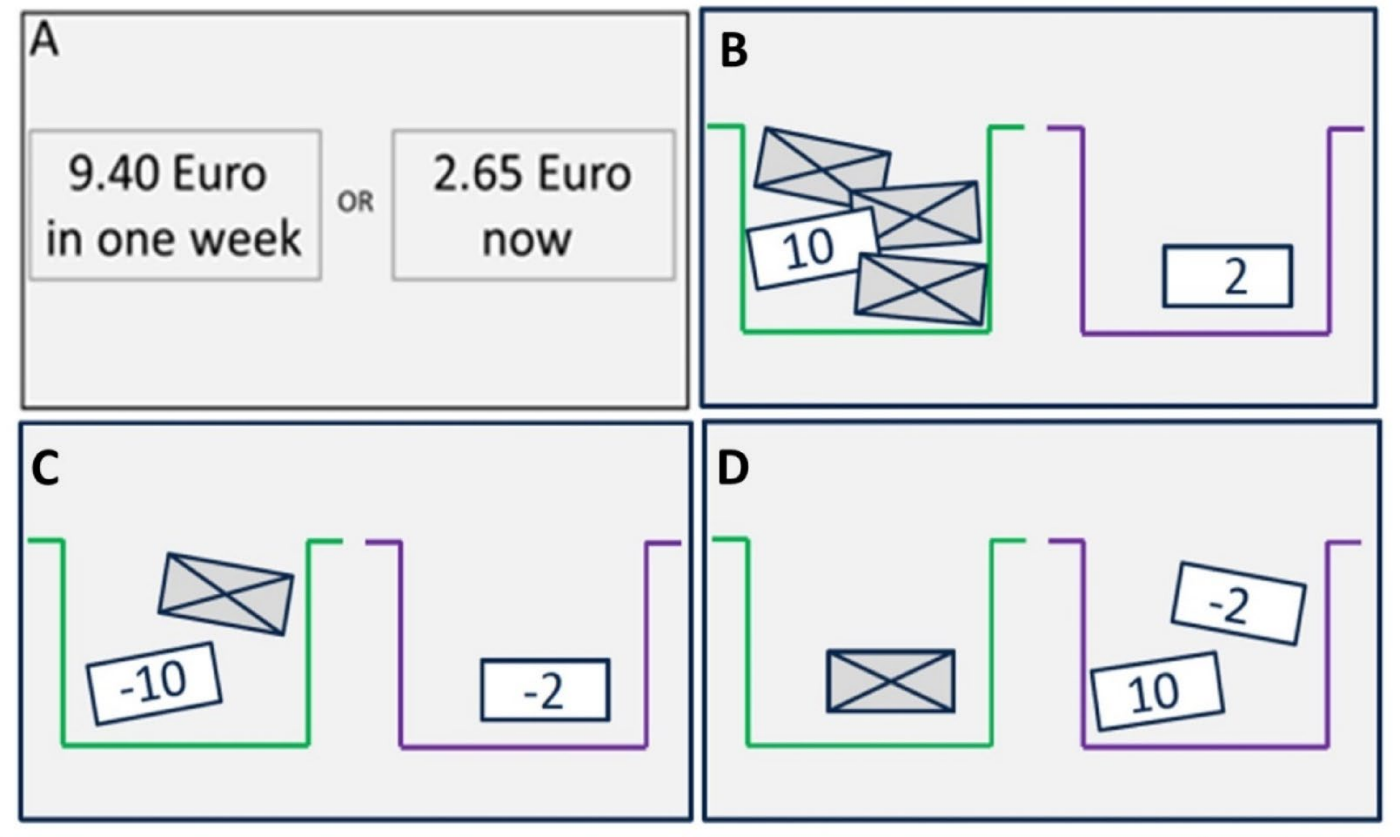
Schematic overview of the tasks in our decision-making battery. **A**) Delay aversion in the delay discounting task, **B**) risk-seeking for gains in the probability discounting for gains task, **C**) risk-seeking for losses in the probability discounting for losses task, and **D**) loss aversion in the mixed gambles task. Note. Reproduced from Kräplin et al. (2020), licensed under CC BY^11^.

Value-based decision-making was measured using four tasks designed by Pooseh et al. (Fig. 2)^10^. All tasks were adaptive, with the options presented on a computer screen using Psychtoolbox in MATLAB (http://psychtoolbox.org). A Bayesian algorithm updated estimates after each trial, providing highly informative choice options near the individual’s indifference point. This method enabled precise inference of decision-making parameters, including the discounting rate (*k*) for delay and probability tasks and the loss aversion parameter (λ (lambda)) for the mixed gambles task. The concrete decision-making facets and tasks are as follows:

1. **Delay aversion in the delay-discounting task**: Participants chose between smaller immediate rewards and larger delayed rewards with delays ranging from 3 to 365 days. A higher *k* value indicates greater delay discounting.

2. **Risk-seeking for gains in the probability discounting for gains task**: Participants made decisions involving uncertain gains, with probabilities ranging from 2/3 to 1/5. A higher *k* value indicates a lower risk-seeking for gains.

3. **Risk-seeking for losses in the probability discounting for losses task**: Participants made decisions involving uncertain losses, with probabilities ranging from 2/3 to 1/5. These first three tasks consisted of 30 trials each and monetary gains/losses ranged from 0.30–10€. A higher *k* value indicates a higher risk-seeking for losses.

4. **Loss aversion in the mixed gambles task**: Participants were presented with gain-loss scenarios to assess loss aversion, starting with 10€ and playing 40 trials with 1–40€ for gains and 5–20€ for losses. A higher λ value indicates higher loss aversion.

We hypothesized that higher *k* values in the delay discounting task (i.e., higher delay aversion), lower *k* values in the probability discounting tasks (i.e., higher risk-seeking for gains and lower risk-seeking for losses), and lower λ values (i.e., lower loss aversion) would increase the likelihood of addictive behavior.

### Covariates

Covariates included age, gender, IQ, and group membership at baseline. Age and gender were assessed during the first diagnostic session. Gender was assessed via self-report using a binary response format (i.e., “male” or “female”), in accordance with the study protocol at the time of data collection (2013–2016). The survey instrument did not capture non-binary gender identities and could therefore not analyze them. IQ was measured using the Hamburg-Wechsler Adult Intelligence Scale-Revised (HAWIE)^33^.

### Statistical analysis

**Fig. 3:**
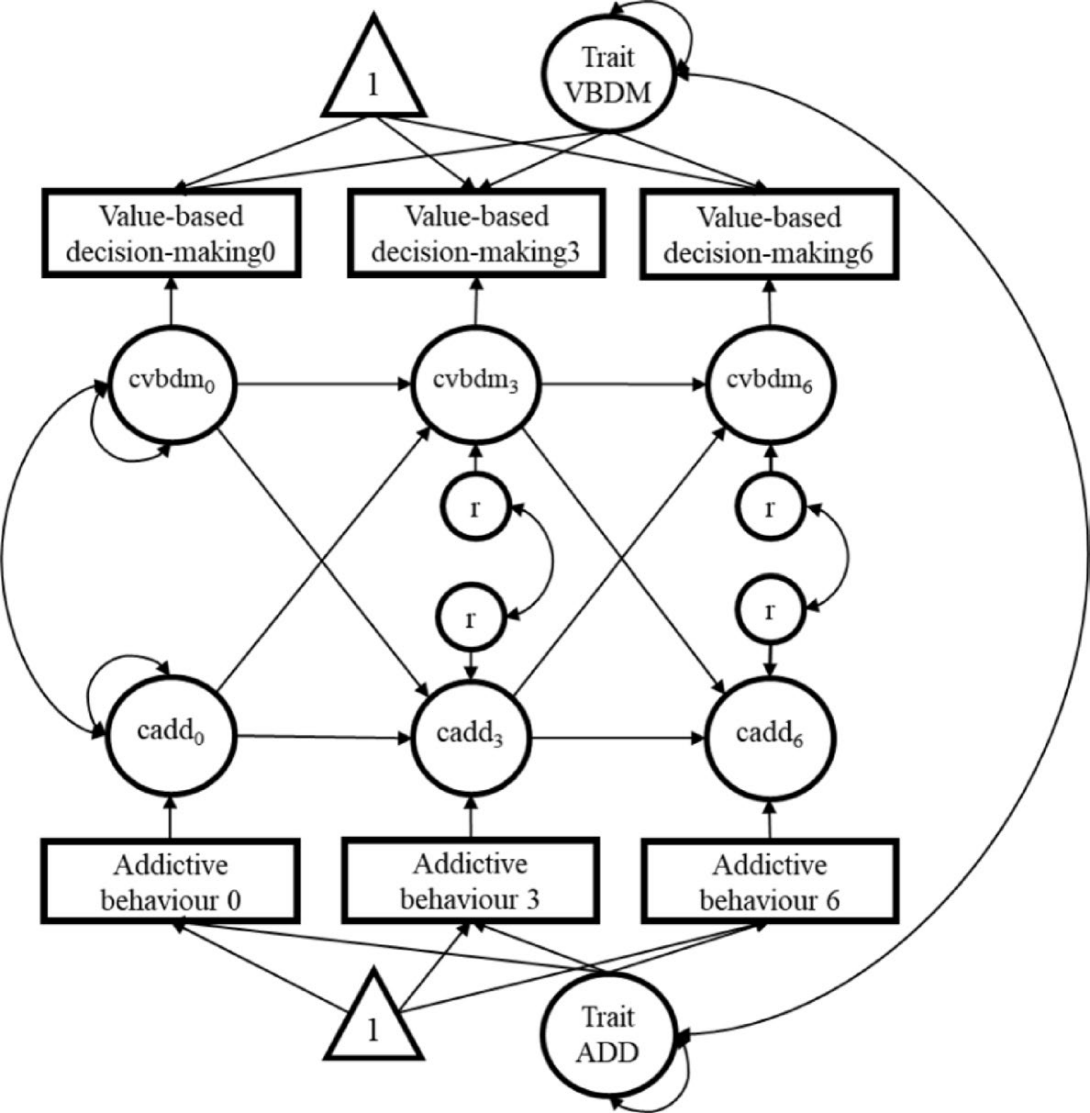
Proposed random intercept cross-lagged panel model (RI-CLPM; Hamaker et al., 2015) for the causal relationship between daily self-control failures and addictive behavior over three time points (baseline, 3-year and 6-year follow-ups). Triangles represent constants (for the mean structure), squares represent observed variables, and circles represent latent variables. Note: vbdm = value-based decision-making (we specified 4 models for each indicator of value-based decision-making: k-values for delay discounting, probability discounting for gains and losses, and lambda for loss aversion). add = addictive behavior (we specified 3 models for each indicator of addictive behavior: quantity of use, frequency of use, and DSM-5 criteria). cvbdm/ cadd = within-person centered variables. r = residual variance of the within-person centered variables. time points are indicated by the numbers: 0 = baseline, 3 = 3-year follow-up, 6 = 6-year follow-up.

To evaluate the bidirectional hypotheses regarding the causal relationships between value-based decision-making and addictive behavior (see preregistration on OSF 10.17605/OSF.IO/BWC46), we implemented RI-CLPM^19^ within the framework of structural equation modeling (SEM). The models were estimated using observed variables and robust maximum likelihood (MLR) in Mplus 8.11 (Muthén & Muthén, 1998–2024). Full information maximum likelihood was used, utilizing all available data. The analysis examined the temporal stability (auto-regressive effects) and the temporal ordering (cross-lagged effects) between value-based decision-making and addictive behavior. Figure 3 shows the proposed model, and the Mplus input for the models with and without covariates can be found on OSF (https://osf.io/y7jek/). Separate models were developed for each of the four non-standardized indicators of value-based decision-making (*k*-value in the delay discounting task and the probability discounting for gains and losses tasks, and λ for loss aversion) and the three non-standardized indicators of addictive behavior (quantity of use, frequency of use, and DSM-5 symptoms). In total, 12 RI-CLPMs were conducted without covariates. Model fit was evaluated using the comparative fit index (CFI), root mean square error of approximation (RMSEA), and standardized root mean square residual (SRMR).

As outlined in previous research^11,30,31^ we included the covariates age, gender, IQ, and group assignment at baseline in subsequent models by regressing all observed variables (at time points 0, 3, and 6) on these covariates. Altogether, 12 RI-CLPMs with covariates were run, resulting in a total of 24 models. We used the models with covariates for statistical inference, but checked whether the models without covariates point in the same direction of inference. Where the models with and without covariates differed in the analyses, we report this in the results section.

In response to reviewer feedback, we conducted additional exploratory group comparisons of baseline demographic and decision-making variables despite the fact that these had not been preregistered. We compared age, IQ, and the four decision-making parameters between groups using one-way ANOVAs and Kruskal–Wallis tests as a nonparametric alternative. If the Kruskal–Wallis test yielded different results, we reported them because they are more robust. We assessed group differences in gender, income, school graduation, and education level using chi-square tests.

## Results

### Sample description and group comparison

The sample characteristics at baseline can be found in Table 1. The majority of our sample consisted of highly educated individuals, many of whom were university students. Exploratory analyses revealed no significant differences between the substance use disorder, behavioral addiction and control groups in terms of baseline age, IQ, gender distribution, income, school graduation or education level (all *p* > 0.05).

The detailed descriptive data for all three waves and each addictive behavior (i.e., alcohol use, tobacco use, internet use, gaming, gambling, and shopping) are presented in Table 2. While the variance in individual addictive behaviors is sometimes low due to the small number of participants with the addictive disorder, the aggregated measure of all addictive behaviors shows meaningful variance. In all three waves, the consumption levels were low to medium and the addictive disorder severity according to the DSM-5 severity specifiers were mainly mild to moderate (Table S1). Although the intention was to include many addictive behaviors to examine transdiagnostic processes in addiction, the sample does not include gambling or shopping addiction.

Table S2 in the supplemental material shows that there was no systematic dropout in the demographic variables or the addictive behavior variables over the three waves, except for IQ, which was lower in the dropouts at the 3-year follow-up (M = 102, SD = 9) compared to completers (M = 105, SD = 10; t = 1.98, *p* = 0.048). Since we controlled for IQ in our analyses, we consider that there is no systematic attrition bias in our study findings.

**Fig. 4:**
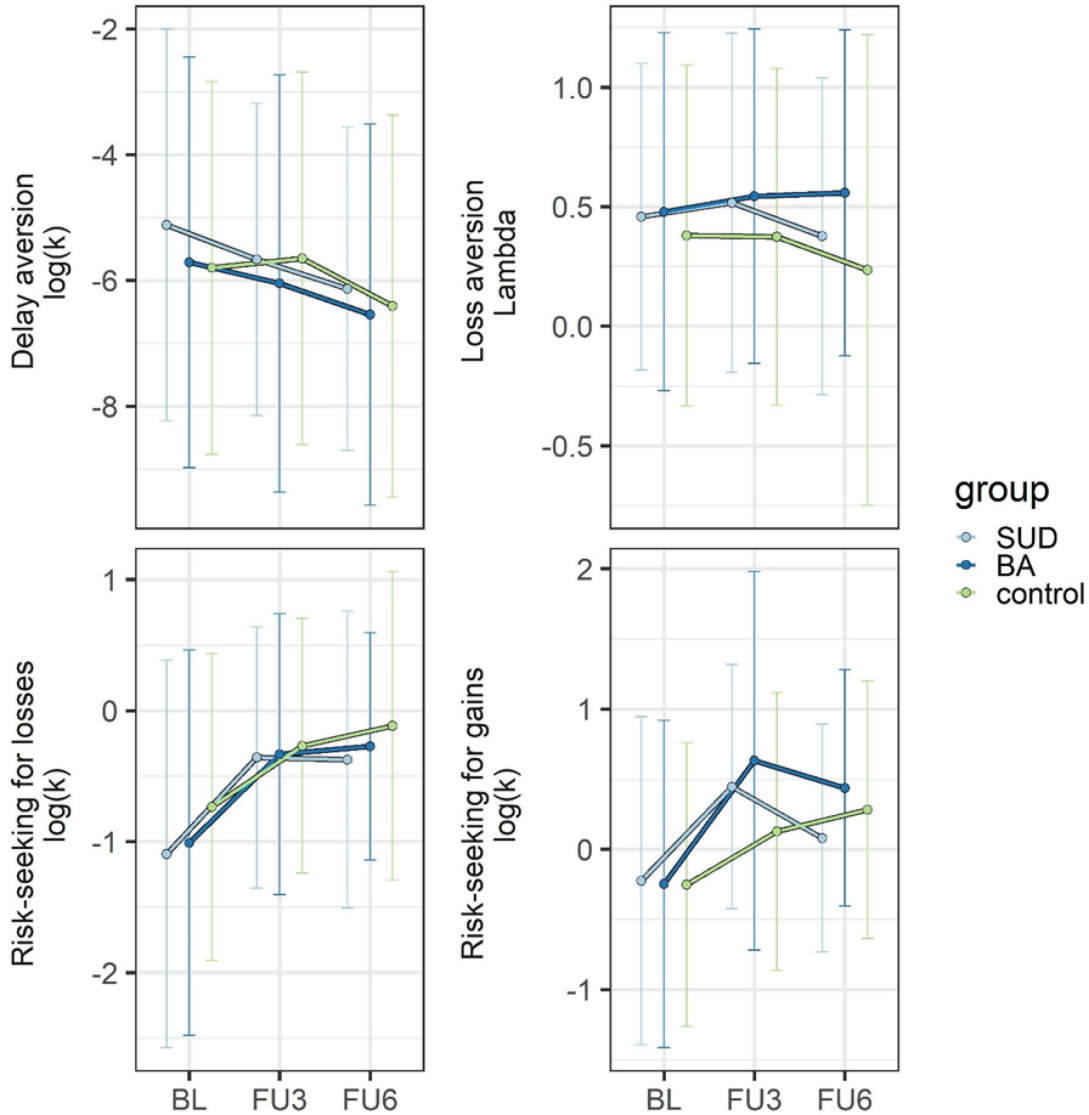
Descriptive overview of the mean values and standard deviations of the decision-making parameters across the three study groups and across all three study waves. Note: SUD = substance use disorder; BA = behavioral addiction; BL = baseline; FU = follow-up.

Descriptive data for the value-based decision tasks across all waves are presented in Fig. 4. Exploratory analyses revealed no significant differences among the substance use disorder, behavioral addiction, and control groups in terms of decision-making parameters (all *p* > 0.05). However, the behavioral addiction group exhibited higher risk-taking for gains than the control group at the 3-year follow-up (F(2,244) = 4.82; *p* = 0.01; η²=0.04; mean difference = 0.50; Bonferroni-corrected post hoc tests, *p* = 0.01). However, due to the exploratory nature of our analyses, the large number of comparisons, and the small effect sizes, this finding should be interpreted with caution.

A correlation matrix between measures of addictive behavior and value-based decision-making for three waves of the study can be found in Table S3 in the supplemental material. Overall, the correlations between these variables were low.

**Table 1:** Demographic characteristics of the baseline sample with means (M) and standard deviations (SD) or numbers (n) and percentages for the substance use disorder (SUD) group, the behavioral addiction (BA) group, and the control group.

Baseline sample	SUD	BA	control	Group comparison
*N* = 338	100	118	120	
	*M (SD)*	*M (SD)*	*M (SD)*	
Age	21.8 (1.6)	21.8 (1.7)	21.9 (1.8)	F(2,335) = 0.33, *p* = 0.72)
Intelligence quotient	103.7 (8.9)	104.4 (10.1)	104.8 (10.4)	F(2,335) = 0.35, *p* = 0.71)
	*n* (%)	*n* (%)	*n* (%)	
Female participants	53 (53.0%)	70 (59.3%)	76 (63.3%)	Chi2 = 2.42, *p* = 0.30
Income ≤ 1000 Euro per month	75 (75.8%)	92 (77.0%)	89 (75.4%)	Chi2 = 6.39, *p* = 0.90
School graduation Gymnasium^a, b^	70 (70.7%)	87 (73.7%)	98 (83.0%)	Chi2 = 5.68, *p* = 0.46
In education, pupils, or students	72 (72.7%)	87 (73.7%)	87 (73.7%)^b^	Chi2 = 0.04, *p* = 0.98

^a^ Gymnasium is considered a type of secondary school in Germany, which qualifies for university entrance.

^b^ Three participants had missing values.

**Table 2:** Descriptive overview of addictive behavior separately for three waves of the study.

	Quantity of use (see first column)			Frequency of use (days per week)			DSM-5 criteria (of the 11 criteria)		
	**Baseline**	**3-year FU**	**6-year FU**	**Baseline**	**3-year FU**	**6-year FU**	**Baseline**	**3-year FU**	**6-year FU**
n	338	252	241	338	252	241	338	252	241
	**Median** **(range)**	**Median** **(range)**	**Median** **(range)**	**Median** **(range)**	**Median** **(range)**	**Median** **(range)**	**Median** **(range)**	**Median** **(range)**	**Median** **(range)**
Tobacco (cigarettes/ day)	0 (0–20)	0 (0–30)	0 (0–20)	0 (0–7)	0 (0–7)	0 (0–7)	0 (0–7)	0 (0–6)	0 (0–6)
Alcohol (g ethanol/ occasion)	36 (0-194)	36 (0-158)	27 (0-171)	0.5 (0–7)	2 (0–7)	0.5 (0–7)	0 (0–6)	0 (0–6)	0 (0–6)
Internet use (hours/ occasion)	1 (0–10)	5 (0–5)	5 (0–5)	4 (0–7)	0.5 (0–7)	0.5 (0–7)	0 (0–9)	0 (0–7)	0 (0–7)
Computer gaming (hours/ occasion)	0 (0–5)	0 (0–5)	1 (0–10)	0 (0–7)	0.25 (0–7)	0.25 (0–7)	0 (0–8)	0 (0–6)	0 (0–4)
Gambling (hours/ occasion)	0 (0–4)	0 (0–1)	0 (0–1)	0 (0–2)	0 (0–4)	0 (0–2)	0 (0–2)	0 (0–2)	0 (0–1)
Shopping (hours/ occasion)	0 (0–3)	0 (0–7)	0 (0–4)	0 (0–2)	0 (0–2)	0 (0–2)	0 (0–1)	0 (0–6)	0 (0–4)
	**Mean (range)**	**Mean (range)**	**Mean (range)**	**Mean (range)**	**Mean (range)**	**Mean (range)**	**Mean (range)**	**Mean (range)**	**Mean (range)**
Standardized and aggregated values^a^	0.37 (0-1.6)	0.74 (0-2.5)	0.79 (0.1–1.6)	6.71 (0.25–21.3)	5.64 (0–20)	5.34 (0.25–21.5)	3.03 (0–16)	2.14 (0–16)	2.03 (0–14)

Note: FU = follow-up.

^a^The possible value ranges for the outcomes were: quantity 0 to 6 (according to the rescaling from 0 to 1 and the 6 addictive behaviors), frequency 0 to 42 (according to the maximum of seven days per week and the 6 addictive behaviors), and DSM-5 criteria 0 to 64 (according to the maximum of 11 DSM-5 criteria (with the exception of gambling disorder with 9 criteria) and the 6 addictive disorders).

### Hypothesis testing

**Fig. 5:**
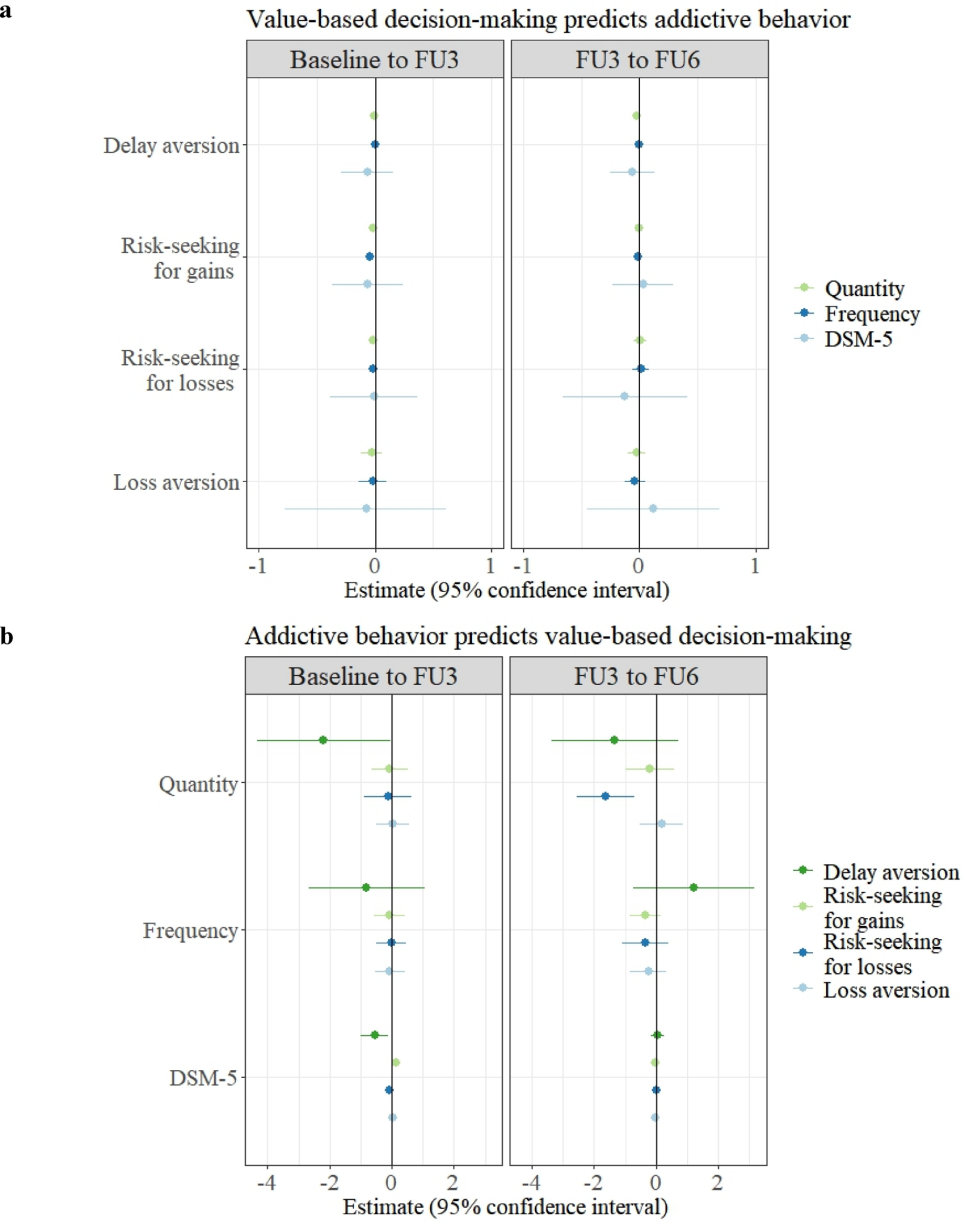
Results of hypothesis testing. Estimates and 95% confidence intervals of the cross-lagged paths from the random intercept cross-lagged panel models (RI-CLPMs) for the evidence of prediction of either (a) value-based decision-making by addictive behavior 3 years ago or (b) addictive behavior by value-based decision-making 3 years ago. Note: FU = follow-up; Quantity = quantity of addictive behavior, Frequency = frequency of addictive behavior, DSM-5 = addictive disorder severity according to the number of met DSM-5 criteria.

The 12 tables with the results of the 12 RI-CLPMs to test our hypotheses are in the supplemental material due to their volume. An overview of the results of the cross-lagged paths, which were the most important paths of the 12 RI-CLPMs for testing our hypotheses, can be found in Fig. 5. For clarity, the results are presented below, first according to the four decision-making parameters and then according to the three addiction outcomes.

### Delay aversion and addictive behavior

#### Quantity of use

The model of the quantity of use and delay aversion with covariates yielded a good model fit (χ2 = 1.428, df = 1, *p* = 0.232, RMSEA = 0.036 [0.000–0.154], CFI = 0.999, SRMR = 0.010). The parameter estimates of the model with covariates are presented in Table S4-1 in the supplemental material. The autoregressive coefficients were significantly positive for quantity of use. This suggests that there is stability in quantity of use beyond the trait-like stability captured by the random intercept. The cross-lagged paths are of interest for our hypothesis testing. Against our hypothesis, the cross-lagged paths from quantity of use at baseline to delay aversion at the 3-year follow-up (β=-2.190, *p* = 0.045; Fig. 5, left side) and from this to the quantity of use at the 6-year follow-up (β=-0.025, *p* = 0.045; Fig. 5, right side) were negative and significant. However, in the model without covariates, these paths were not significant (β=-1.894, *p* = 0.07 and β=-0.023, *p* = 0.07, respectively). Given this covariate effect and that the hypothesis predicted a positive relationship, this finding will not be further interpreted.

#### Frequency of use

The model with covariates yielded an adequate to good model fit (χ2 = 3.909, df = 1, *p* = 0.048, RMSEA = 0.093 [0.007–0.197], CFI = 0.992, SRMR = 0.012). The parameter estimates of the model with covariates are presented in Table S4-2 in the supplemental material. The autoregressive coefficients were significantly positive for frequency of use. All other paths were not significant (see also Fig. 4 for the cross-lagged paths).

#### Addictive disorder severity

The model with covariates yielded a good model fit (χ2 = 0.354, df = 1, *p* = 0.552, RMSEA = 0.000 [0.000–0.121], CFI = 1.000, SRMR = 0.004). The parameter estimates of the model with covariates are presented in Table S4-3 in the supplemental material. Only the autoregressive path from the number of met DSM-5 criteria at the 3-year follow-up to the number of DSM-5 criteria at the 6-year follow-up was significantly positive. The cross-lagged path from the number of met DSM-5 criteria at baseline to delay aversion at the 3-year follow-up was significantly negative (β=-0.545, *p* = 0.018; see also Fig. 4b). Given that the hypothesis predicted a positive relationship, this finding will not be further interpreted.

Overall, we found no significant correlations among the trait components indicating that there is no evidence for a relationship between higher delay aversion and addictive behavior at the between-person level. At the within-person level, we found that many of the autoregressive effects are positive and statistically significant for addictive behavior. This indicates that persons who exhibit increased addictive behavior at one time point are more likely to exhibit increased addictive behavior at the next time point. The cross-lagged effects are the focus of hypothesis testing in this study. All cross-lagged paths show no evidence to support the hypothesis that an increase in delay aversion within an individual predicts a future increase in addictive behavior or vice versa.

### Risk-seeking for gains and addictive behavior

#### Quantity of use

The model with covariates yielded a good model fit (χ2 = 9.006, df = 5, *p* = 0.109, RMSEA = 0.049 [0.000–0.099], CFI = 0.983, SRMR = 0.023). The parameter estimates of the model with covariates are presented in Table S5-1 in the supplemental material. The autoregressive coefficients were significantly positive for quantity of use and risk-seeking for gains. All other paths were not significant (see also Fig. 4 for the cross-lagged paths).

#### Frequency of use

The model with covariates yielded a good model fit (χ = 0.689, df = 2, *p* = 0.709, RMSEA = 0.000 [0.000–0.079], CFI = 1.000, SRMR = 0.005). The parameter estimates of the model with covariates are presented in Table S5-2 in the supplemental material. The autoregressive coefficients were significantly positive for frequency of use and risk-seeking for gains. One cross-lagged path showed that higher risk-seeking for gains at baseline (i.e., a lower *k* value) predicts a higher frequency of use at the 3-year follow-up (β=-0.044, *p* = 0.007), which supported our hypothesis (see also Fig. 4a). The size of the effect was small. None of the other paths was significant. In the model without covariates, we found a significantly negative cross-lagged path from risk-seeking for gains at the 3-year follow-up to frequency of use at the 6-year follow-up (β=-0.089, *p* = 0.045).

#### Addictive disorder severity

The model with covariates yielded a good model fit (χ2 = 0.016, df = 1, *p* = 0.898, RMSEA = 0.000 [0.000–0.065], CFI = 1.000, SRMR = 0.001). The parameter estimates of the model with covariates are presented in Table S5-3 in the supplemental material. Only the autoregressive paths from risk-seeking for gains and the number of met DSM-5 criteria at the 3-year follow-up to the 6-year follow-up were significantly positive. All other paths were not significant (see also Fig. 4 for the cross-lagged paths).

Overall, we found no evidence for a relationship between higher risk-seeking for gains and addictive behavior at the between-person level. At the within-person level, we found that persons who display higher risk-seeking for gains or increased addictive behavior at one time point are more likely to have a higher score on the same variable at the next time point. Concerning our hypotheses, we found almost no evidence that an increase in an individual’s risk-seeking for gains predicts a future increase in addictive behavior or vice versa. A significant, albeit small, effect was that higher risk-seeking for gains at baseline predicted higher frequency of use at the 3-year follow-up.

### Risk-seeking for losses and addictive behavior

#### Quantity of use

The model with covariates yielded a good model fit (χ2 = 0.267, df = 1, *p* = 0.605, RMSEA = 0.000 [0.000–0.115], CFI = 1.000, SRMR = 0.005). The parameter estimates of the model with covariates are presented in Table S6-1 in the supplemental material. The autoregressive coefficients were significantly positive for quantity of use. One cross-lagged path indicated that a higher quantity of use at the 3-year follow-up significantly predicted a lower risk-seeking for losses (i.e., lower *k* values) at the 6-year follow-up (β=-1.635, *p* = 0.001; see also Fig. 4b), which partly supported our hypothesis. The size of the effect was small. All other paths were not significant.

#### Frequency of use

The model with covariates yielded a good model fit (χ2 = 2.947, df = 2, *p* = 0.229, RMSEA = 0.037 [0.000–0.121], CFI = 0.997, SRMR = 0.015). The parameter estimates of the model with covariates are presented in Table S6-2 in the supplemental material. The autoregressive coefficients were significantly positive for frequency of use. All other paths were not significant (see also Fig. 4 for the cross-lagged paths).

#### Addictive disorder severity

The model with covariates yielded a good model fit (χ2 = 0.053, df = 1, *p* = 0.817, RMSEA = 0.000 [0.000–0.088], CFI = 1.000, SRMR = 0.002). The parameter estimates of the model with covariates are presented in Table S6-3 in the supplemental material. At the between-person level, the relationship between the random intercept factors was significantly positive, but small (β = 0.205, *p* = 0.049). Against our hypothesis, more risk-seeking for losses was associated with more DSM-5 criteria met, but the between-person correlations do not capture within-person variation over time and therefore do not indicate directionality. Therefore, the cross-lagged paths are of interest for our hypothesis testing. However, only the autoregressive path from the number of DSM-5 criteria met at the 3-year follow-up to the 6-year follow-up was significantly positive. All other paths were not significant (Fig. 4).

Overall, we found no evidence for a relationship between higher risk-seeking for losses and addictive behavior at the between-person level. At the within-person level, we found that persons who display higher risk-seeking for losses or increased addictive behavior at one time point are more likely to have a higher score on the same variable at the next time point. Concerning our hypotheses, we found no evidence that a decrease in risk-seeking for losses within an individual predicts a future increase in addictive behavior or vice versa.

### Loss aversion and addictive behavior

#### Quantity of use

The model with covariates yielded a good model fit (χ2 = 0.014, df = 1, *p* = 0.907, RMSEA = 0.000 [0.000–0.061], CFI = 1.000, SRMR = 0.001). The parameter estimates of the model with covariates are presented in Table S7-1 in the supplemental material. Only the autoregressive path from quantity of use at the 3-year follow-up to quantity of use at the 6-year follow-up was significantly positive. All other paths were not significant (see also Fig. 4 for the cross-lagged paths).

#### Frequency of use

The model with covariates yielded a good model fit (χ2 = 0.096, df = 1, *p* = 0.757, RMSEA = 0.000 [0.000–0.098], CFI = 1.000, SRMR = 0.003). The parameter estimates of the model with covariates are presented in Table S7-2 in the supplemental material. The autoregressive coefficients were significantly positive for frequency of use. All other paths were not significant (Fig. 4).

#### Addictive disorder severity

The model with covariates yielded a good model fit (χ2 = 0.880, df = 2, *p* = 0.644, RMSEA = 0.000 [0.000–0.085], CFI = 1.000, SRMR = 0.006). The parameter estimates of the model with covariates are presented in Table S7-3 in the supplemental material. Only the autoregressive path from the number of met DSM-5 criteria at the 3-year follow-up to the 6-year follow-up was significantly positive. All other paths were not significant (Fig. 4).

Overall, we found no evidence for a relationship between lower loss aversion and addictive behavior at the between-person level. At the within-person level, we found that persons who display higher loss aversion or increased addictive behavior at one time point are also more likely to have a higher score on the same variable at the next time point. Concerning our hypotheses, we found no evidence that a lower loss aversion within an individual predicts a future increase in addictive behavior or vice versa.

## Discussion

Our study examined the longitudinal and bidirectional relationships between four facets of value-based decision-making (delay aversion, risk-seeking for gains, risk-seeking for losses, and loss aversion) and addictive behavior, operationalized as quantity and frequency of use and DSM-5-defined severity. Using RI-CLPMs, we are one of the first studies to disentangle within-person dynamics from stable between-person differences. While previous research has highlighted altered value-based decision-making as central to addictive behavior, our findings provide a nuanced view of the interplay between these factors over time.

We found that both decision-making parameters and addictive behaviors were mostly stable over time, as indicated by significant autoregressive effects in most RI-CLPMs. Contrary to our initial hypotheses, we found little evidence that changes in value-based decision-making predict future addictive behaviors, or vice versa. To enhance transparency, we note that we did not apply corrections for multiple comparisons because our conclusions are based on overall patterns of results rather than isolated significant effects. These patterns show that altered value-based decision-making does not affect later addictive behavior, and vice versa. We also observed limited evidence of a cross-sectional relationship between altered decision-making and addictive behavior. The sample characteristics, the assessment, and the size of the effect may explain these null findings.

First, our sample was young and highly educated. Most participants showed low levels of consumption and only mild to moderate addictive disorder severity. This differs from the typical composition of clinical addiction samples in previous research. This difference may have (i) limited our ability to detect cross-sectional or longitudinal relationships due to smaller effect sizes, and (ii) reduced the generalizability of our findings. Regarding (i) effect sizes, our sample was underrepresented by more severe cases of addictive disorders, who may exhibit stronger impairments in value-based decision-making^1^. Regarding (ii) generalizability, our results are not generalizable to clinical samples with lower functioning. However, it is worth noting that clinical samples have limitations as well. For example, fewer than 10% of individuals with alcohol use disorder seek treatment^34^. Therefore, no single sampling approach can fully capture the heterogeneity of addictive behavior. Multiple complementary research approaches are needed to gain a more comprehensive understanding of different population segments. In future studies, it would be important to achieve a more representative sample with respect to these parameters. This could be achieved, for example, through randomized recruitment in different educational institutions using demographic strata and by specifically recruiting typically underrepresented groups, e.g., those with less education.

Second, a relatively high proportion of the individuals in our sample identified as female. Although our models controlled for gender, we acknowledge that gender-related differences in addiction vulnerability are well-established. For instance, men have a prevalence rate of AUD that is five times higher than that of women^35^. While we recognize that sex and gender may influence addiction trajectories, our sample size was too small to allow for disaggregated RI-CLPMs. Future studies should aim to collect larger, gender-balanced samples to enable such analyses. Additionally, gender was assessed using a binary format and participants could not indicate a non-binary identity. This reflects limitations in past data collection practices. Future research should adopt more inclusive and precise assessments of gender identity.

Third, the decision-making tasks and the interviews to assess the aggregated addictive behavior may not be sensitive enough to capture subtle within-person changes or interactions over time, particularly if only a subset of individuals show substantial variation in either predictor or outcome variables. Future studies may apply ambulatory assessment such as Ecological Momentary Assessment (EMA) to assess daily consumption^36^or gamified tasks to assess daily decision making^37^. This would enable us to study more fine-grained temporal dynamics of decision-making and addictive behavior.

Fourth, the effect size of the longitudinal relationship between altered decision-making and addictive behavior may be smaller than we expected in our power analyses based on moderate effect sizes (see Sect. 1 of the supplemental material). Small effect sizes may be due to the multifaceted nature of addiction, where altered value-based decision-making is only one of many contributing factors, along with genetic predisposition, environmental and social factors. For example, in another longitudinal study using the large IMAGEN cohort (baseline *n* = 2,220), we found that higher delay aversion significantly predicted more alcohol use problems, but this explained only 1% of the variance in alcohol development^17^.

Fifth, although we wanted to investigate transdiagnostic mechanisms, we did not include all addictive behaviors. Some behaviors, such as the use of illicit substances, were not included at all, while others, such as gambling or shopping, were included but not captured by the recruitment strategy. While transdiagnostic conclusions may be drawn from the four addictive behaviors studied, previous research suggests that different addictive behaviors, such as alcohol and internet use, may involve distinct cognitive and neurobiological mechanisms^38,39^. However, due to the relatively low prevalence of specific addictive behaviors in our sample, we could not conduct behavior-specific RI-CLPMs. On the one hand, a transdiagnostic approach enabled us to identify shared variance across four addiction-related behaviors, which may reflect common underlying processes. On the other hand, future studies may benefit from separating substance-related and non-substance-related behaviors, or from focusing on specific types of addiction with adequate base rates.

Alternatively, our findings may represent a genuine absence of a causal relationship between value-based decision-making and addictive behavior. At first glance, this appears to challenge previous studies suggesting that steeper delay discounting robustly predicts substance use initiation and escalation^1,16^. For example, in previous studies from our lab, we observed that greater delay aversion, lower risk-seeking for losses, and reduced loss aversion were significant predictors of different addictive behaviors^11,12^. Thus, while altered value-based decision-making has been proposed as a transdiagnostic marker of addiction^20^ our findings suggest that its influence may not be as directly causal or consistent over time and severity stages as previously thought. These findings are consistent with criticisms of the overgeneralization of specific decision-making alterations as universal causes of addiction^40^. This interpretation would suggest that although altered decision-making and addictive behaviors are correlated cross-sectionally or even longitudinally, they do not exert a strong causal influence on each other over time. Instead, these constructs may emerge independently as manifestations of common underlying factors, such as neurobiological vulnerabilities or environmental influences. For example, both impulsive decision-making and addiction severity may be driven by common predispositions, such as deficits in executive functioning or heightened sensitivity to reward, without directly influencing each other longitudinally.

In summary, our findings suggest that altered value-based decision-making and addictive behaviors may not have simple causal relationships in a highly educated, non-clinical sample Along with previous correlational studies^20,21^, our findings imply that value-based decision-making may be a transdiagnostic marker of addiction vulnerability rather than a mechanism that causes changes in addictive behavior over time. This distinction has important implications for applied settings. Specifically, altered value-based decision-making could facilitate the early identification of individuals at risk for developing addictive behaviors and inform prevention efforts. For example, screening tools that evaluate delay aversion or loss sensitivity could identify individuals at risk before they engage in problematic use. However, given the apparent lack of predictive effects within individuals in our study, interventions targeting altered decision-making alone may be insufficient for treating addictive behaviors. Our findings further underscore the importance of more longitudinal within-person studies considering situational factors such as stress, environment, and emotional states. Regarding stress, it has been shown to exacerbate impulsive decision-making and increase vulnerability to addiction by dysregulating brain systems related to reward and executive function^41^. In terms of environmental factors, peer norms and social rewards can reinforce risky decision-making, especially during adolescence^42^. Negative emotional states, such as anxiety or depression, bias decisions toward short-term rewards^21^. These dynamics suggest that alterations in value-based decision-making may have stronger effects on addictive behavior in specific contexts, emphasizing the need for longitudinal studies using EMA to capture real-life behavior and interactions. Our findings highlight the importance of using rigorous causal testing methods in addiction research. They suggest that previously assumed causal links between decision-making and addiction may be overstated, and call for a re-evaluation of theoretical models. Future research should focus on identifying the conditions under which decision-making impairments contribute to addiction risk, rather than assuming a direct predictive relationship. This highlights the need for a broader theoretical framework that integrates additional factors such as individual differences in vulnerability to addiction and contextual factors^6,43^. These models should then be tested in well-powered studies on more diverse groups. These groups should include individuals with various addictive behaviors, clinical samples, and individuals from underrepresented backgrounds.

## Supplementary Information

Below is the link to the electronic supplementary material.

Supplementary Material 1

## Data Availability

The data and the analysis input files for Mplus are available on the Open Science Framework (OSF) at https://osf.io/y7jek/.
