# Supplementary material for: Risk, reward and loss in addictive behavior: a six-year cross-lagged panel study
Source: Sci Rep. 2025 Aug 30;15:31958. doi: 10.1038/s41598-025-17826-0 (PMC12398614; doi:10.1038/s41598-025-17826-0)
Supplement: Supplementary file 1 — Supplementary Material 1 [file 41598_2025_17826_MOESM1_ESM.docx]

**Supplemental material to:**

**Risk, reward and loss in addictive behavior: A six-year longitudinal study of decision-making dynamics**

**Anja Kräplin^1,2^*, Mohsen Joshanloo^3^, Juliane Hilde Fröhner^1^, Christian Baeuchl^1^, Gerhard Bühringer^2,4^, Thomas Goschke^2^, Michael N. Smolka^1^**

^1^Department of Psychiatry and Psychotherapy, Technische Universität Dresden, Germany

^2^Faculty of Psychology, Technische Universität Dresden, Germany

^3^Department of Psychology, Keimyung University, Daegu, South Korea

^4^IFT Institut für Therapieforschung, Munich, Germany

*** Correspondence:**Anja Kräplin

[anja.kraeplin@tu-dresden.de](mailto:anja.kraeplin@tu-dresden.de)

Keywords: value-based decision-making, impulsivity, addictive behaviors, longitudinal, cross-lagged panel

Content

[1. Sample size calculation 5](#_Toc200443343)

[2. Sample description 6](#_Toc200443344)

[Table S1: Severity of substance-related and additive disorders (AD) according to the DSM-5 specifiers at baseline and follow-up (1 year later) separately for the substance use disorder (SUD) group, the non-substance-related addictive disorder (ND) group, and the control group. 6](#_Toc200443345)

[Table S2. Results of the group difference tests of baseline sample characteristics between completers and the dropouts at the 3-years follow-up (FU3) and the 6-year follow-up (FU6) in terms of addictive behavior (symptoms, quantity, frequency) and the control variables age, gender, IQ, and group membership at baseline. 8](#_Toc200443346)

[Table S3. Correlation matrix between measures of addictive behavior and value-based decision-making for three waves of the study. 9](#_Toc200443347)

[3. Detailed results of the RI-CLPMs for hypothesis testing 10](#_Toc200443348)

[Table S4-1. Parameter estimates for the random intercept cross‐lagged panel models (RI-CLPM) including delay aversion (delay discounting task, DD) and quantity of addictive behavior. 10](#_Toc200443349)

[Table S4-2. Parameter estimates for the random intercept cross‐lagged panel models (RI-CLPM) including delay aversion (delay discounting task, DD) and frequency of addictive behavior. 11](#_Toc200443350)

[Table S4-3. Parameter estimates for the random intercept cross‐lagged panel models (RI-CLPM) including delay aversion (delay discounting task, DD) and addictive disorder severity. 12](#_Toc200443351)

[Table S5-1. Parameter estimates for the random intercept cross‐lagged panel models (RI-CLPM) including risk-seeking for gains (probability discounting of gains task, PDG) and quantity of addictive behavior. 13](#_Toc200443352)

[Table S5-2. Parameter estimates for the random intercept cross‐lagged panel models (RI-CLPM) including risk-seeking for gains (probability discounting of gains task, PDG) and frequency of addictive behavior. 14](#_Toc200443353)

[Table S5-3. Parameter estimates for the random intercept cross‐lagged panel models (RI-CLPM) including risk-seeking for gains (probability discounting of gains task, PDG) and addictive disorder severity. 15](#_Toc200443354)

[Table S6-1. Parameter estimates for the random intercept cross‐lagged panel models (RI-CLPM) including risk-seeking for losses (probability discounting of losses task, PDL) and quantity of addictive behavior. 16](#_Toc200443355)

[Table S6-2. Parameter estimates for the random intercept cross‐lagged panel models (RI-CLPM) including risk-seeking for losses (probability discounting of losses task, PDL) and frequency of addictive behavior. 17](#_Toc200443356)

[Table S6-3. Parameter estimates for the random intercept cross‐lagged panel models (RI-CLPM) including risk-seeking for losses (probability discounting of losses task, PDL)and addictive disorder severity. 18](#_Toc200443357)

[Table S7-1. Parameter estimates for the random intercept cross‐lagged panel models (RI-CLPM) including loss aversion (mixed gambles task, MG) and quantity of addictive behavior. 19](#_Toc200443358)

[Table S7-2. Parameter estimates for the random intercept cross‐lagged panel models (RI-CLPM) including loss aversion (mixed gambles task, MG) and frequency of addictive behavior. 20](#_Toc200443359)

[Table S7-3. Parameter estimates for the random intercept cross‐lagged panel models (RI-CLPM) including loss aversion (mixed gambles task, MG) and addictive disorder severity. 21](#_Toc200443360)

# 1. Sample size calculation

The sample size for the whole project was estimated using Stata 13 for multiple linear regression with power (1- β) = .80, significance level α= 0.05, and five covariates (age, gender, IQ, income, school graduation). We assumed moderate group differences at baseline with R^2^=.05 according to previous studies comparing individuals with gambling disorder, alcohol dependence, and Tourette syndrome on measures of executive functions and decision-making (Goudriaan et al. 2005; Goudriaan et al. 2006). The necessary sample size would have been N=235 in total. Furthermore, we assumed a dropout rate of 30% during the first funding phase of the project (3 years). The final estimated sample size was 330 with 110 in each group.

# 2. Sample description

## Table S1: Severity of additive disorders according to the DSM-5 specifiers at baseline separately for the substance use disorder (SUD) group, the behavior addiction (BA) group, and the control group.

|  | **SUD**  **n=100** | **ND**  **n=118** | **Controls**  **n=120** |
| --- | --- | --- | --- |
| DSM-5 SUD  Tobacco-related  Mild  Moderate  Severe  Alcohol-related  Mild  Moderate  Severe | n=61  53%  33%  14%  n=55  84%  11%  5% | n=0  n=0 | n=0  n=0 |
| (Adapted) DSM-5 BA  Internet-related  Mild  Moderate  Severe  Gaming-related  Mild  Moderate  Severe  Gambling-related  Mild  Moderate  Severe  Shopping-related  Mild  Moderate  Severe | n=0  n=0  n=0  n=0 | n= 101  61%  31%  8%  n=34  44%  32%  24%  n=1  100%  0%  0%  n=0 | n=0  n=0  n=0  n=0 |

## Table S2. Results of the group difference tests of baseline sample characteristics between completers and the dropouts at the 3-year follow-up (FU3) and the 6-year follow-up (FU6) in terms of addictive behavior (symptoms, quantity, frequency) and the control variables age, gender, IQ, and group membership at baseline.

|  | Completers at FU3 | Dropouts at FU3 | Test statistics (completers vs. dropouts FU3) | Completers at FU6 | Dropouts at FU6 | Test statistics  (completers vs. dropouts FU6) |
| --- | --- | --- | --- | --- | --- | --- |
| Total n=338 | 253 (75%) | 85 (25%) |  | 242 (72%) | 96 (28%) |  |
| Addictive behavior at baseline |  |  |  |  |  |  |
| Quantity | 0.37 (0.26) | 0.40 (0.26) | *t*=-1.22, *p*=0.22 | 0.36 (0.25) | 0.42 (0.26) | *t*=-1.88, *p*=.06 |
| Frequency | 6.61 (4.30) | 7.03 (4.88) | *t*=-0.75, *p*=0.45 | 6.45 (4.27) | 7.38 (4.83) | *t*=-1.73, *p*=0.09 |
| DSM-5 criteria | 2.92 (2.66) | 3.38 (2.93) | *z*=-1.12, *p*=0.29 | 2.84 (2.65) | 3.53(2.87) | *z*=-1.12, *p*=0.26 |
| Control variables at baseline |  |  |  |  |  |  |
| Age | 21.82 (1.77) | 21.89 (1.58) | *t*=-0.35, *p*=0.73 | 21.86 (1.77) | 21.78 (1.59) | *t*=0.38, *p*=0.71 |
| Female gender | 147 (44%) | 52 (61%) | χ2=0.25, *p*=0.62 | 143 | 56 | χ2=0.02, *p*=0.90 |
| IQ | 105.01 (9.94) | 102.57 (9.44) | ***t*=1.98, *p*=0.048** | 104.90 (10.16) | 102.12 (9.00) | *t*=1.50, *p*=0.13 |
| Group at baseline  SUD  ND  Controls | 69 (27%)  89 (35%)  95 (38%) | 31 (37%)  29 (34%)  25 (29%) | χ2=3.03, *p*=0.22 | 64 (26%)  84 (35%)  94 (39%) | 36 (38%)  34 (35%)  26 (27%) | χ2=5.53, *p*=0.06 |

*Note:* We have computed parametric tests for metric data (e.g. t-test), but also non-parametric methods (e.g. Wilcoxon rank sum test) in case distributional assumptions are violated. Where the results differ, we report the results of the robust non-parametric methods.

**Bold** **p**-**values** **indicate** statistical significance at the 5% level.

## Table S3. Correlation matrix between measures of addictive behavior and value-based decision-making for three waves of the study.

|  | **Baseline** | | | | **3-year FU** | | | | **6-year FU** | | | |
| --- | --- | --- | --- | --- | --- | --- | --- | --- | --- | --- | --- | --- |
|  | DD | PDG | PDL | MG | DD | PDG | PDL | MG | DD | PDG | PDL | MG |
| **Baseline** |  |  |  |  |  |  |  |  |  |  |  |  |
| Quantity | 0.093 | 0.072 | 0.067 | -0.090 | -0.085 | 0.037 | 0.072 | 0.004 | 0.004 | -0.091 | 0.024 | 0.001 |
| Frequency | 0.016 | 0.049 | 0.058 | 0.056 | -0.062 | 0.021 | 0.026 | 0.052 | 0.051 | -0.016 | -0.034 | 0.074 |
| DSM-5 | 0.042 | 0.025 | -0.010 | 0.042 | -0.112 | **0.191** | 0.006 | **0.126** | -0.010 | 0.038 | 0.005 | 0.134 |
| **3-year FU** |  |  |  |  |  |  |  |  |  |  |  |  |
| Quantity | 0.097 | -0.061 | -0.005 | -0.084 | 0.001 | -0.001 | -0.029 | 0.021 | -0.014 | -0.089 | **-0.237** | 0.084 |
| Frequency | 0.085 | **-0.161** | -0.037 | -0.034 | -0.006 | 0.009 | 0.019 | -0.005 | 0.127 | -0.124 | -0.034 | -0.086 |
| DSM-5 | 0.025 | -0.079 | 0.021 | -0.016 | -0.019 | 0.029 | 0.091 | -0.024 | 0.085 | -0.074 | 0.09 | -0.001 |
| **6-year FU** |  |  |  |  |  |  |  |  |  |  |  |  |
| Quantity | **0.140** | **0.151** | 0.037 | -0.103 | -0.039 | -0.051 | 0.073 | -0.094 | -0.029 | -0.070 | 0.029 | -0.132 |
| Frequency | -0.009 | **-0.152** | 0.055 | -0.081 | -0.029 | -0.085 | 0.083 | -0.090 | 0.053 | -0.113 | 0.009 | -0.094 |
| DSM-5 | 0.022 | -0.034 | 0.075 | -0.064 | -0.041 | 0.005 | 0.073 | -0.025 | 0.050 | -0.045 | -0.018 | 0.021 |

*Note:* FU=follow-up; DD=delay aversion (delay discounting task); PDG=risk-seeking for gains (probability discounting of gains task); PDL=risk-seeking for losses (probability discounting of losses task; MG=loss aversion (mixed gambles task, MG); Quantity=quantity of addictive behavior, Frequency=frequency of addictive behavior, DSM-5=addictive disorder severity according to the number of met DSM-5 criteria

**Bold** **p**-**values** **indicate** statistical significance at the 5% level.

# 3. Detailed results of the RI-CLPMs for hypothesis testing

## Table S4-1. Parameter estimates for the random intercept cross‐lagged panel models (RI-CLPM) including delay aversion (delay discounting task, DD) and quantity of addictive behavior.

|  |  |  |  | |  | | 95% Confidence interval | |  | |
| --- | --- | --- | --- | --- | --- | --- | --- | --- | --- | --- |
| Predictor |  | Outcome | Unstandardized  coefficient | p | | Lower | | Upper | | Standardized coefficient |
|  | Autoregressive paths | |  |  | |  | |  | |  |
| DD 0 | → | DD 3 | 0.108 | 0.402 | | -0.144 | | 0.360 | | 0.116 |
| DD 3 | → | DD 6 | 0.214 | 0.103 | | -0.043 | | 0.471 | | 0.223 |
| Quantity 0 | → | Quantity 3 | 0.318 | **0.011** | | 0.074 | | 0.561 | | 0.286 |
| Quantity 3 | → | Quantity 6 | 0.198 | **0.021** | | 0.029 | | 0.367 | | 0.203 |
|  | Cross-lagged paths | |  |  | |  | |  | |  |
| DD 0 | → | Quantity 3 | -0.006 | 0.540 | | -0.027 | | 0.014 | | -0.062 |
| DD 3 | → | Quantity 6 | -0.025 | **0.045** | | -0.048 | | -0.001 | | -0.230 |
| Quantity 0 | → | DD 3 | -2.190 | **0.045** | | -4.334 | | -0.045 | | -0.216 |
| Quantity 3 | → | DD 6 | -1.337 | 0.199 | | -3.376 | | 0.703 | | -0.152 |
|  | Covariance (between) | |  |  | |  | |  | |  |
| Trait DD | ↔ | Trait Quantity | 0.061 | 0.266 | | -0.047 | | 0.169 | | 0.727 |

*Note.* The model includes the covariates age, gender, IQ, and group membership at baseline. **Bold** **p**-**values** **indicate** statistical significance in the hypothesized direction at the 5% level.

## Table S4-2. Parameter estimates for the random intercept cross‐lagged panel models (RI-CLPM) including delay aversion (delay discounting task, DD) and frequency of addictive behavior.

|  |  |  |  | |  | | 95% Confidence interval | |  | |
| --- | --- | --- | --- | --- | --- | --- | --- | --- | --- | --- |
| Predictor |  | Outcome | Unstandardized  coefficient | p | | Lower | | Upper | | Standardized coefficient |
|  | Autoregressive paths | |  |  | |  | |  | |  |
| DD 0 | → | DD 3 | 0.142 | 0.296 | | -0.124 | | 0.408 | | 0.154 |
| DD 3 | → | DD 6 | 0.259 | 0.054 | | -0.004 | | 0.523 | | 0.273 |
| Frequency 0 | → | Frequency 3 | 0.278 | **0.024** | | 0.037 | | 0.520 | | 0.289 |
| Frequency 3 | → | Frequency 6 | 0.411 | **0.000** | | 0.182 | | 0.640 | | 0.387 |
|  | Cross-lagged paths | |  |  | |  | |  | |  |
| DD 0 | → | Frequency 3 | 0.007 | 0.585 | | -0.018 | | 0.031 | | 0.063 |
| DD 3 | → | Frequency 6 | -0.003 | 0.796 | | -0.025 | | 0.019 | | -0.023 |
| Frequency 0 | → | DD 3 | -0.804 | 0.398 | | -2.667 | | 1.059 | | -0.098 |
| Frequency 3 | → | DD 6 | 1.220 | 0.219 | | -0.724 | | 3.165 | | 0.150 |
|  | Covariance (between) | |  |  | |  | |  | |  |
| Trait DD | ↔ | Trait Frequency | -0.008 | 0.903 | | -0.132 | | 0.116 | | -0.117 |

*Note.* The model includes the covariates age, gender, IQ, and group membership at baseline. **Bold** **p**-**values** **indicate** statistical significance in the hypothesized direction at the 5% level.

## Table S4-3. Parameter estimates for the random intercept cross‐lagged panel models (RI-CLPM) including delay aversion (delay discounting task, DD) and addictive disorder severity.

|  |  |  |  | |  | | 95% Confidence interval | |  | |
| --- | --- | --- | --- | --- | --- | --- | --- | --- | --- | --- |
| Predictor |  | Outcome | Unstandardized  coefficient | p | | Lower | | Upper | | Standardized coefficient |
|  | Autoregressive paths | |  |  | |  | |  | |  |
| DD 0 | → | DD 3 | 0.084 | 0.539 | | -0.185 | | 0.354 | | 0.092 |
| DD 3 | → | DD 6 | 0.243 | 0.054 | | -0.004 | | 0.490 | | 0.257 |
| Severity 0 | → | Severity 3 | -0.050 | 0.846 | | -0.550 | | 0.451 | | -0.033 |
| Severity 3 | → | Severity 6 | 0.334 | **0.000** | | 0.162 | | 0.505 | | 0.376 |
|  | Cross-lagged paths | |  |  | |  | |  | |  |
| DD 0 | → | Severity 3 | -0.067 | 0.554 | | -0.288 | | 0.154 | | -0.071 |
| DD 3 | → | Severity 6 | -0.064 | 0.510 | | -0.253 | | 0.126 | | -0.070 |
| Severity 0 | → | DD 3 | -0.545 | **0.018** | | -0.995 | | -0.095 | | -0.373 |
| Severity 3 | → | DD 6 | 0.045 | 0.675 | | -0.165 | | 0.255 | | 0.049 |
|  | Covariance (between) | |  |  | |  | |  | |  |
| Trait DD | ↔ | Trait Severity | 0.472 | 0.181 | | -0.219 | | 1.162 | | 0.191 |

*Note.* The model includes the covariates age, gender, IQ, and group membership at baseline. **Bold** **p**-**values** **indicate** statistical significance in the hypothesized direction at the 5% level.

## Table S5-1. Parameter estimates for the random intercept cross‐lagged panel models (RI-CLPM) including risk-seeking for gains (probability discounting of gains task, PDG) and quantity of addictive behavior.

|  |  |  |  | |  | | 95% Confidence interval | |  | |
| --- | --- | --- | --- | --- | --- | --- | --- | --- | --- | --- |
| Predictor |  | Outcome | Unstandardized  coefficient | p | | Lower | | Upper | | Standardized coefficient |
|  | Autoregressive paths | |  |  | |  | |  | |  |
| PDG 0 | → | PDG 3 | 0.260 | **0.035** | | 0.019 | | 0.501 | | 0.266 |
| PDG 3 | → | PDG 6 | 0.277 | **0.012** | | 0.061 | | 0.493 | | 0.354 |
| Quantity 0 | → | Quantity 3 | 0.271 | **0.045** | | 0.006 | | 0.537 | | 0.241 |
| Quantity 3 | → | Quantity 6 | 0.196 | **0.027** | | 0.023 | | 0.370 | | 0.201 |
|  | Cross-lagged paths | |  |  | |  | |  | |  |
| PDG 0 | → | Quantity 3 | -0.015 | 0.366 | | -0.049 | | 0.018 | | -0.072 |
| PDG 3 | → | Quantity 6 | -0.001 | 0.944 | | -0.024 | | 0.022 | | -0.004 |
| Quantity 0 | → | PDG 3 | -0.060 | 0.842 | | -0.651 | | 0.531 | | -0.012 |
| Quantity 3 | → | PDG 6 | -0.206 | 0.601 | | -0.979 | | 0.566 | | -0.057 |
|  | Covariance (between) | |  |  | |  | |  | |  |
| Trait PDG | ↔ | Trait Quantity | 0.000 | 999.000 | | 0.000 | | 0.000 | | 0.000 |

*Note.* The model includes the covariates age, gender, IQ, and group membership at baseline. **Bold** **p**-**values** **indicate** statistical significance in the hypothesized direction at the 5% level.

## Table S5-2. Parameter estimates for the random intercept cross‐lagged panel models (RI-CLPM) including risk-seeking for gains (probability discounting of gains task, PDG) and frequency of addictive behavior.

|  |  |  |  | |  | | 95% Confidence interval | |  | |
| --- | --- | --- | --- | --- | --- | --- | --- | --- | --- | --- |
| Predictor |  | Outcome | Unstandardized  coefficient | p | | Lower | | Upper | | Standardized coefficient |
|  | Autoregressive paths | |  |  | |  | |  | |  |
| PDG 0 | → | PDG 3 | 0.280 | **0.022** | | 0.040 | | 0.519 | | 0.286 |
| PDG 3 | → | PDG 6 | 0.308 | **0.002** | | 0.113 | | 0.504 | | 0.389 |
| Frequency 0 | → | Frequency 3 | 0.297 | **0.014** | | 0.061 | | 0.533 | | 0.308 |
| Frequency 3 | → | Frequency 6 | 0.419 | **0.000** | | 0.197 | | 0.642 | | 0.396 |
|  | Cross-lagged paths | |  |  | |  | |  | |  |
| PDG 0 | → | Frequency 3 | -0.044 | **0.007** | | -0.076 | | -0.012 | | -0.187 |
| PDG 3 | → | Frequency 6 | -0.013 | 0.288 | | -0.036 | | 0.011 | | -0.049 |
| Frequency 0 | → | PDG 3 | -0.075 | 0.771 | | -0.580 | | 0.430 | | -0.019 |
| Frequency 3 | → | PDG 6 | -0.335 | 0.183 | | -0.829 | | 0.158 | | -0.102 |
|  | Covariance (between) | |  |  | |  | |  | |  |
| Trait PDG | ↔ | Trait Frequency | 0.000 | 999.000 | | 0.000 | | 0.000 | | 0.000 |

*Note.* The model includes the covariates age, gender, IQ, and group membership at baseline. **Bold** **p**-**values** **indicate** statistical significance in the hypothesized direction at the 5% level.

## Table S5-3. Parameter estimates for the random intercept cross‐lagged panel models (RI-CLPM) including risk-seeking for gains (probability discounting of gains task, PDG) and addictive disorder severity.

|  |  |  |  | |  | | 95% Confidence interval | |  | |
| --- | --- | --- | --- | --- | --- | --- | --- | --- | --- | --- |
| Predictor |  | Outcome | Unstandardized  coefficient | p | | Lower | | Upper | | Standardized coefficient |
|  | Autoregressive paths | |  |  | |  | |  | |  |
| PDG 0 | → | PDG 3 | 0.244 | 0.056 | | -0.006 | | 0.494 | | 0.250 |
| PDG 3 | → | PDG 6 | 0.277 | **0.009** | | 0.069 | | 0.485 | | 0.353 |
| Severity 0 | → | Severity 3 | -0.084 | 0.745 | | -0.593 | | 0.424 | | -0.056 |
| Severity 3 | → | Severity 6 | 0.323 | **0.000** | | 0.151 | | 0.495 | | 0.366 |
|  | Cross-lagged paths | |  |  | |  | |  | |  |
| PDG 0 | → | Severity 3 | -0.062 | 0.690 | | -0.364 | | 0.241 | | -0.030 |
| PDG 3 | → | Severity 6 | 0.030 | 0.822 | | -0.232 | | 0.292 | | 0.016 |
| Severity 0 | → | PDG 3 | 0.122 | 0.077 | | -0.013 | | 0.257 | | 0.169 |
| Severity 3 | → | PDG 6 | -0.015 | 0.568 | | -0.067 | | 0.037 | | -0.040 |
|  | Covariance (between) | |  |  | |  | |  | |  |
| Trait PDG | ↔ | Trait Severity | -0.054 | 0.647 | | -0.285 | | 0.177 | | -0.173 |

*Note.* The model includes the covariates age, gender, IQ, and group membership at baseline. **Bold** **p**-**values** **indicate** statistical significance in the hypothesized direction at the 5% level.

## Table S6-1. Parameter estimates for the random intercept cross‐lagged panel models (RI-CLPM) including risk-seeking for losses (probability discounting of losses task, PDL) and quantity of addictive behavior.

|  |  |  |  | |  | | 95% Confidence interval | |  | |
| --- | --- | --- | --- | --- | --- | --- | --- | --- | --- | --- |
| Predictor |  | Outcome | Unstandardized  coefficient | p | | Lower | | Upper | | Standardized coefficient |
|  | Autoregressive paths | |  |  | |  | |  | |  |
| PDL 0 | → | PDL 3 | -0.042 | 0.580 | | -0.190 | | 0.107 | | -0.065 |
| PDL 3 | → | PDL 6 | -0.044 | 0.837 | | -0.469 | | 0.380 | | -0.040 |
| Quantity 0 | → | Quantity 3 | 0.254 | **0.044** | | 0.006 | | 0.501 | | 0.226 |
| Quantity 3 | → | Quantity 6 | 0.194 | **0.019** | | 0.031 | | 0.357 | | 0.199 |
|  | Cross-lagged paths | |  |  | |  | |  | |  |
| PDL 0 | → | Quantity 3 | -0.013 | 0.496 | | -0.049 | | 0.024 | | -0.062 |
| PDL 3 | → | Quantity 6 | 0.008 | 0.789 | | -0.051 | | 0.067 | | 0.026 |
| Quantity 0 | → | PDL 3 | -0.127 | 0.747 | | -0.902 | | 0.647 | | -0.036 |
| Quantity 3 | → | PDL 6 | -1.635 | **0.001** | | -2.557 | | -0.713 | | -0.469 |
|  | Covariance (between) | |  |  | |  | |  | |  |
| Trait PDL | ↔ | Trait Quantity | 0.012 | 0.409 | | -0.017 | | 0.041 | | 0.247 |

*Note.* The model includes the covariates age, gender, IQ, and group membership at baseline. **Bold** **p**-**values** **indicate** statistical significance in the hypothesized direction at the 5% level.

## Table S6-2. Parameter estimates for the random intercept cross‐lagged panel models (RI-CLPM) including risk-seeking for losses (probability discounting of losses task, PDL) and frequency of addictive behavior.

|  |  |  |  | |  | | 95% Confidence interval | |  | |
| --- | --- | --- | --- | --- | --- | --- | --- | --- | --- | --- |
| Predictor |  | Outcome | Unstandardized  coefficient | p | | Lower | | Upper | | Standardized coefficient |
|  | Autoregressive paths | |  |  | |  | |  | |  |
| PDL 0 | → | PDL 3 | -0.045 | 0.636 | | -0.232 | | 0.142 | | -0.070 |
| PDL 3 | → | PDL 6 | 0.026 | 0.916 | | -0.451 | | 0.502 | | 0.023 |
| Frequency 0 | → | Frequency 3 | 0.277 | **0.031** | | 0.025 | | 0.528 | | 0.287 |
| Frequency 3 | → | Frequency 6 | 0.402 | **0.001** | | 0.170 | | 0.634 | | 0.378 |
|  | Cross-lagged paths | |  |  | |  | |  | |  |
| PDL 0 | → | Frequency 3 | -0.018 | 0.261 | | -0.048 | | 0.013 | | -0.078 |
| PDL 3 | → | Frequency 6 | 0.014 | 0.695 | | -0.057 | | 0.085 | | 0.038 |
| Frequency 0 | → | PDL 3 | -0.020 | 0.937 | | -0.511 | | 0.471 | | -0.007 |
| Frequency 3 | → | PDL 6 | -0.358 | 0.345 | | -1.101 | | 0.385 | | -0.116 |
|  | Covariance (between) | |  |  | |  | |  | |  |
| Trait PDL | ↔ | Trait Frequency | 0.000 | 999.000 | | 0.000 | | 0.000 | | 0.000 |

*Note.* The model includes the covariates age, gender, IQ, and group membership at baseline. **Bold** **p**-**values** **indicate** statistical significance in the hypothesized direction at the 5% level.

## Table S6-3. Parameter estimates for the random intercept cross‐lagged panel models (RI-CLPM) including risk-seeking for losses (probability discounting of losses task, PDL) and addictive disorder severity.

|  |  |  |  | |  | | 95% Confidence interval | |  | |
| --- | --- | --- | --- | --- | --- | --- | --- | --- | --- | --- |
| Predictor |  | Outcome | Unstandardized  coefficient | p | | Lower | | Upper | | Standardized coefficient |
|  | Autoregressive paths | |  |  | |  | |  | |  |
| PDL 0 | → | PDL 3 | -0.061 | 0.435 | | -0.214 | | 0.092 | | -0.096 |
| PDL 3 | → | PDL 6 | 0.008 | 0.974 | | -0.449 | | 0.464 | | 0.007 |
| Severity 0 | → | Severity 3 | -0.073 | 0.782 | | -0.586 | | 0.441 | | -0.048 |
| Severity 3 | → | Severity 6 | 0.327 | **0.000** | | 0.150 | | 0.503 | | 0.373 |
|  | Cross-lagged paths | |  |  | |  | |  | |  |
| PDL 0 | → | Severity 3 | -0.009 | 0.961 | | -0.383 | | 0.365 | | -0.005 |
| PDL 3 | → | Severity 6 | -0.126 | 0.643 | | -0.661 | | 0.408 | | -0.046 |
| Severity 0 | → | PDL 3 | -0.072 | 0.344 | | -0.220 | | 0.077 | | -0.148 |
| Severity 3 | → | PDL 6 | -0.010 | 0.800 | | -0.091 | | 0.070 | | -0.029 |
|  | Covariance (between) | |  |  | |  | |  | |  |
| Trait PDL | ↔ | Trait Severity | 0.205 | **0.049** | | 0.001 | | 0.410 | | 0.230 |

*Note.* The model includes the covariates age, gender, IQ, and group membership at baseline. **Bold** **p**-**values** **indicate** statistical significance in the hypothesized direction at the 5% level.

## Table S7-1. Parameter estimates for the random intercept cross‐lagged panel models (RI-CLPM) including loss aversion (mixed gambles task, MG) and quantity of addictive behavior.

|  |  |  |  | |  | | 95% Confidence interval | |  | |
| --- | --- | --- | --- | --- | --- | --- | --- | --- | --- | --- |
| Predictor |  | Outcome | Unstandardized  coefficient | p | | Lower | | Upper | | Standardized coefficient |
|  | Autoregressive paths | |  |  | |  | |  | |  |
| MG 0 | → | MG 3 | 0.106 | 0.589 | | -0.278 | | 0.489 | | 0.105 |
| MG 3 | → | MG 6 | 0.194 | 0.190 | | -0.096 | | 0.485 | | 0.165 |
| Quantity 0 | → | Quantity 3 | 0.234 | 0.068 | | -0.018 | | 0.485 | | 0.208 |
| Quantity 3 | → | Quantity 6 | 0.184 | **0.023** | | 0.025 | | 0.344 | | 0.189 |
|  | Cross-Lagged paths | |  |  | |  | |  | |  |
| MG 0 | → | Quantity 3 | -0.029 | 0.542 | | -0.121 | | 0.063 | | -0.067 |
| MG 3 | → | Quantity 6 | -0.022 | 0.543 | | -0.095 | | 0.050 | | -0.054 |
| Quantity 0 | → | MG 3 | 0.033 | 0.901 | | -0.496 | | 0.563 | | 0.013 |
| Quantity 3 | → | MG 6 | 0.174 | 0.620 | | -0.514 | | 0.863 | | 0.063 |
|  | Covariance (between) | |  |  | |  | |  | |  |
| Trait MG | ↔ | Trait Quantity | 0.001 | 0.959 | | -0.021 | | 0.022 | | 0.016 |

*Note.* The model includes the covariates age, gender, IQ, and group membership at baseline. **Bold** **p**-**values** **indicate** statistical significance in the hypothesized direction at the 5% level.

## Table S7-2. Parameter estimates for the random intercept cross‐lagged panel models (RI-CLPM) including loss aversion (mixed gambles task, MG) and frequency of addictive behavior.

|  |  |  |  | |  | | 95% Confidence interval | |  | |
| --- | --- | --- | --- | --- | --- | --- | --- | --- | --- | --- |
| Predictor |  | Outcome | Unstandardized  coefficient | p | | Lower | | Upper | | Standardized coefficient |
|  | Autoregressive paths | |  |  | |  | |  | |  |
| MG 0 | → | MG 3 | 0.118 | 0.551 | | -0.270 | | 0.506 | | 0.117 |
| MG 3 | → | MG 6 | 0.198 | 0.160 | | -0.078 | | 0.475 | | 0.169 |
| Frequency 0 | → | Frequency 3 | 0.268 | **0.031** | | 0.025 | | 0.511 | | 0.278 |
| Frequency 3 | → | Frequency 6 | 0.395 | **0.001** | | 0.167 | | 0.623 | | 0.372 |
|  | Cross-Lagged paths | |  |  | |  | |  | |  |
| MG 0 | → | Frequency 3 | -0.020 | 0.729 | | -0.136 | | 0.095 | | -0.043 |
| MG 3 | → | Frequency 6 | -0.037 | 0.421 | | -0.126 | | 0.053 | | -0.073 |
| Frequency 0 | → | MG 3 | -0.059 | 0.809 | | -0.538 | | 0.420 | | -0.029 |
| Frequency 3 | → | MG 6 | -0.259 | 0.383 | | -0.840 | | 0.322 | | -0.104 |
|  | Covariance (between) | |  |  | |  | |  | |  |
| Trait MG | ↔ | Trait Frequency | 0.010 | 0.529 | | -0.021 | | 0.041 | | 0.460 |

*Note.* The model includes the covariates age, gender, IQ, and group membership at baseline. **Bold** **p**-**values** **indicate** statistical significance in the hypothesized direction at the 5% level.

## Table S7-3. Parameter estimates for the random intercept cross‐lagged panel models (RI-CLPM) including loss aversion (mixed gambles task, MG) and addictive disorder severity.

|  |  |  |  | |  | | 95% Confidence interval | |  | |
| --- | --- | --- | --- | --- | --- | --- | --- | --- | --- | --- |
| Predictor |  | Outcome | Unstandardized  coefficient | p | | Lower | | Upper | | Standardized coefficient |
|  | Autoregressive paths | |  |  | |  | |  | |  |
| MG 0 | → | MG 3 | 0.098 | 0.629 | | -0.299 | | 0.495 | | 0.097 |
| MG 3 | → | MG 6 | 0.197 | 0.193 | | -0.100 | | 0.495 | | 0.167 |
| Severity 0 | → | Severity 3 | -0.079 | 0.757 | | -0.582 | | 0.424 | | -0.053 |
| Severity 3 | → | Severity 6 | 0.330 | **0.000** | | 0.156 | | 0.503 | | 0.375 |
|  | Cross-Lagged paths | |  |  | |  | |  | |  |
| MG 0 | → | Severity 3 | -0.07 | 0.825 | | -0.769 | | 0.614 | | -0.019 |
| MG 3 | → | Severity 6 | 0.122 | 0.672 | | -0.444 | | 0.689 | | 0.034 |
| Severity 0 | → | MG 3 | 0.047 | 0.193 | | -0.024 | | 0.119 | | 0.129 |
| Severity 3 | → | MG 6 | -0.019 | 0.467 | | -0.070 | | 0.032 | | -0.066 |
|  | Covariance (between) | |  |  | |  | |  | |  |
| Trait MG | ↔ | Trait Severity | 0.000 | 999.000 | | 0.000 | | 0.000 | | 0.000 |

*Note.* The model includes the covariates age, gender, IQ, and group membership at baseline. **Bold** **p**-**values** **indicate** statistical significance in the hypothesized direction at the 5% level.
